# A Convergence Model of Bioelectric, Gap Junctional, and Hippo–YAP Signalling in Oral Cancer Stem Cell Maintenance

**DOI:** 10.3390/ijms27156649

**Published:** 2026-07-25

**Authors:** Surendra Kumar Acharya, Wei Cheong Ngeow, Firdaus Hariri, Fong Fong Liew, Yee Fan Choon

**Affiliations:** 1 Department of Oral and Maxillofacial Clinical Sciences, Faculty of Dentistry, University of Malaya, Kuala Lumpur 50603, Malaysia; ngeowy@um.edu.my (W.C.N.); firdaushariri@um.edu.my (F.H.); 2 Department of Pharmaceutical Life Sciences, Faculty of Pharmacy, University of Malaya, Kuala Lumpur 50603, Malaysia; jaslff@gmail.com; 3 Department of Pediatric Dentistry, Faculty of Dental Medicine, Universitas Airlangga, Surabaya 60132, Indonesia; choonyeefan@yahoo.com

**Keywords:** bioelectric signalling, cancer stem cells, FAT1, gap junctional intercellular communication, Hippo–YAP signalling, oral squamous cell carcinoma, oral submucous fibrosis

## Abstract

Cancer stem cell (CSC) persistence drives recurrence and therapy resistance in oral squamous cell carcinoma (OSCC), but what keeps cells locked in this stem-like state is poorly understood. In this narrative review, we propose that CSC state is sustained not by any single pathway but by joint dysregulation of three interacting cell-biological systems: membrane potential (V_mem_), communication between neighbouring cells through gap junctional intercellular communication (GJIC), and the Hippo–YAP pathway. We argue that these systems act together on one common point—the YAP protein, retained in the nucleus—which switches on a *SOX2*-centred stemness gene programme and stabilises a self-reinforcing CSC state. Drawing on evidence from cancer genomics, developmental bioelectricity, connexin biology, and OSCC-specific studies, we reconstruct how membrane depolarisation, loss of gap junction coupling, FAT1 mutation, and Hippo pathway inactivation could converge on persistent nuclear YAP, and how betel quid—the principal risk factor across South and Southeast Asia—may engage all three systems at once. Because the model holds that each input reinforces the others, it predicts that targeting several together should displace CSC state more durably than targeting any one alone. We set out the testable predictions this framework generates.

## 1. Introduction

Oral squamous cell carcinoma (OSCC) remains a major cause of cancer-related morbidity worldwide, with recurrence and therapy resistance as persistent clinical challenges [1,2]. Cancer stem cells (CSCs)—a subpopulation endowed with self-renewal, tumorigenic potential, and therapy resistance—are central mediators of these failures [3,4]. The classical hierarchical CSC model, first demonstrated in acute myeloid leukaemia and extended to head and neck squamous cell carcinoma (HNSCC) [5], posited a genetically fixed stem apex generating non-tumorigenic progeny. This model cannot account for the phenotypic plasticity now documented across multiple systems: non-CSC populations regenerate CSC fractions after depletion, CSC states interconvert, and hybrid epithelial–mesenchymal configurations arise stochastically [6,7]. In OSCC specifically, such plasticity correlates with poor prognosis [8,9]. These observations are better accommodated by a systems biology framework in which CSC identity is not a lineage-fixed trait but a dynamic transcriptional state—one of several stable configurations, or attractors, within a high-dimensional gene regulatory landscape [10,11]. Malignant transformation, in this view, involves not merely the accumulation of somatic mutations but a disruption of the regulatory logic governing cell state transitions, locking cells into stem-like attractor basins that are self-reinforcing and difficult to exit.

The Hippo signalling pathway, a conserved regulator of epithelial homeostasis and cell fate, has emerged as a potent driver of CSC maintenance in OSCC. Genomic studies and genome-wide CRISPR fitness screens have identified YAP1 and TAZ, the transcriptional effectors of Hippo, as fitness genes in OSCC cell lines [12,13], and YAP activation has been mechanistically linked to *SOX2*-driven stemness programmes [14,15]. Yet Hippo–YAP does not operate in isolation: recent evidence positions it downstream of mechanosensory inputs, including transmembrane voltage gradients, with membrane depolarisation favouring YAP nuclear retention through a mechano-electro-osmotic mechanism [16]. Bioelectric signalling—mediated through membrane potential (V_mem_), ion channel dynamics, and gap junctional intercellular communication (GJIC)—has emerged as a higher-order regulator of cell fate in both developmental and cancer biology [17,18]. Epithelial tissues maintain coherent bioelectric states through GJIC, through which individual cells integrate into collective mechanosensing fields; disruption of this electrical coordination has been associated with loss of differentiation control and cancer initiation [19]. These mechanisms are particularly relevant in betel quid-associated OSCC, which predominates across South and Southeast Asia. Betel quid induces oral submucous fibrosis (OSF) characterised by extracellular matrix (ECM) stiffening that activates Piezo1-mediated YAP nuclear translocation [20]; arecoline, its principal alkaloid, drives ROS generation, fibrosis, and EMT while directly upregulating YAP/TAZ in oral tissue compartments [21,22,23], positioning Hippo–YAP dysregulation as a functional consequence of betel quid carcinogenesis across multiple cell compartments.

**Figure 1:**
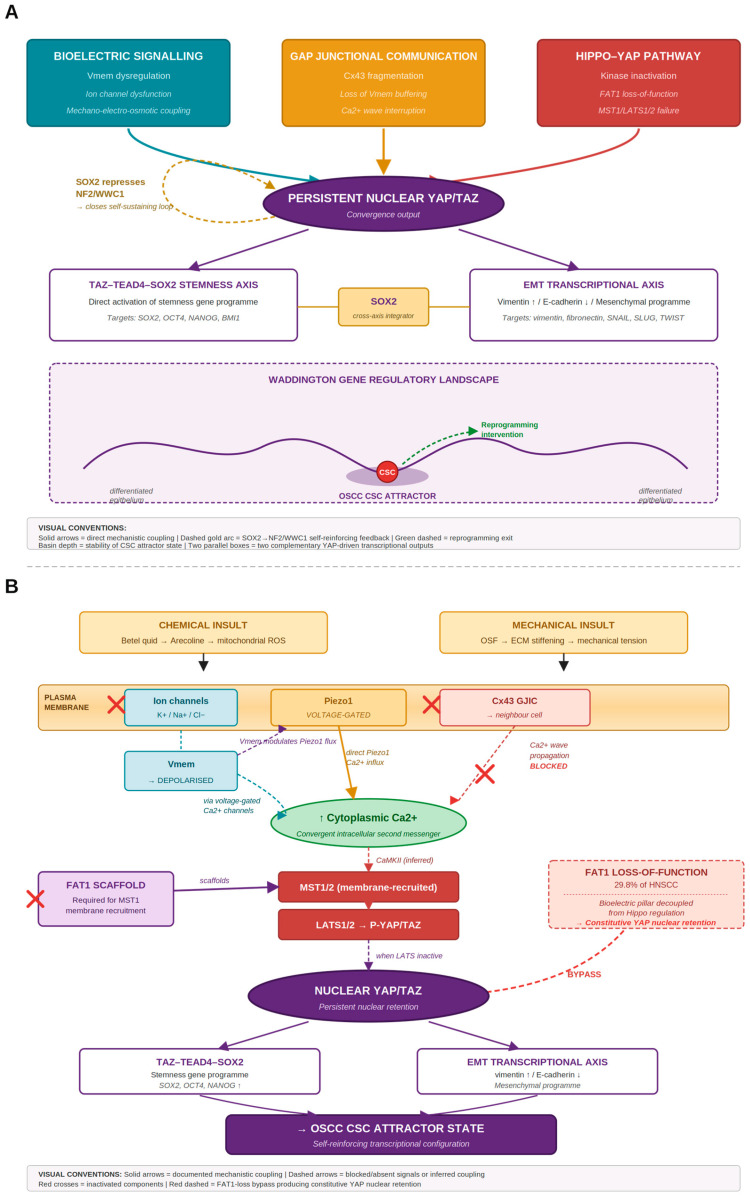
Hippo–YAP as the convergence node of bioelectric and gap junctional signalling in OSCC cancer stem cell attractor stabilisation. (**A**) Three biophysical regulatory systems—tissue bioelectric signalling (V_mem_ dysregulation, teal), gap junctional intercellular communication fragmentation (GJIC, amber), and Hippo–YAP pathway inactivation (coral)—converge on a single regulatory output: nuclear YAP/TAZ. Persistent nuclear YAP/TAZ drives the *TAZ–TEAD4–SOX2* gene regulatory network [14], stabilising oral squamous epithelial progenitor cells in a self-reinforcing CSC attractor state (Waddington landscape). The SOX2 → NF2/WWC1 feedback loop (gold) closes the self-sustaining circuit. Basin depth reflects the stability of the CSC state; the green escape arrow indicates the reprogramming intervention point. (**B**) Molecular cascade in a single epithelial cell. Betel quid alkaloids and ECM stiffening activate Piezo1, shifting cellular biomass density and membrane potential (V_mem_) via mechano-electro-osmotic coupling. GJIC fragmentation (Cx43 downregulation, Cx43–ZO-1 interaction disruption) blocks intercellular Ca^2+^ wave propagation and putative CaMKII-mediated MST1 activation; PTPN14–ZO-1 scaffold disruption further reduces LATS1/2 activity. FAT1 loss-of-function eliminates the Hippo signalling scaffold required for MST1 membrane recruitment. Triple convergence on MST1/2 inactivation → LATS1/2 failure → unphosphorylated YAP/TAZ nuclear retention → *TAZ–TEAD4–SOX2* GRN activation → CSC attractor state. Dashed elements indicate absent or blocked signals; X marks indicate inactivated components.

In this narrative review, we propose that CSC persistence in OSCC is stabilised by the convergent dysregulation of three interconnected biophysical regulatory systems: tissue-level bioelectric signalling (V_mem_), gap junctional intercellular communication (GJIC), and the Hippo–YAP pathway. We argue that these systems do not act independently but converge on a single molecular output—persistent nuclear YAP—which activates the *TAZ–TEAD4–SOX2* gene regulatory network and locks oral epithelial progenitor cells into a self-reinforcing CSC attractor state. Drawing on evidence from genomics, developmental bioelectricity, connexin biology, and OSCC-specific studies, we reconstruct the mechanistic links between V_mem_ depolarisation, Cx43-mediated GJIC fragmentation, FAT1 loss-of-function, and Hippo kinase inactivation, and examine how betel quid exposure simultaneously engages all three dysregulatory axes. The framework generates specific, falsifiable experimental predictions and positions YAP–TEAD-directed combination therapy—targeting the convergence node while co-disrupting upstream biophysical inputs—as a rational strategy for durable CSC state reprogramming rather than cytotoxic elimination (Figure 1).

## 2. Methods

This narrative review was developed to construct and evaluate a mechanistic hypothesis rather than to provide an exhaustive systematic survey, and the literature was therefore identified through a purposive, theme-structured search as below.

Block A (disease): (“oral squamous cell carcinoma” OR OSCC OR “head and neck squamous cell carcinoma” OR HNSCC OR “oral submucous fibrosis” OR OSF).

Block B (Hippo–YAP): (“Hippo pathway” OR YAP OR YAP1 OR TAZ OR WWTR1 OR TEAD OR LATS OR FAT1).

Block C (bioelectric): (“membrane potential” OR V_mem_ OR bioelectric OR “ion channel” OR Piezo1 or mechanotransduction”).

Block D (GJIC): (“gap junction” OR connexin OR Cx43 OR GJA1 OR GJIC).

Block E (CSC): (“cancer stem cell” OR stemness OR SOX2 OR “tumour-initiating cell”) searched individually and in pairwise or three-way combinations (e.g., A AND B AND C) in PubMed/MEDLINE and Google Scholar, English-language, to March 2026. As the central aim was to assess whether these traditionally separate fields intersect, particular attention was given to pairwise and three-way combinations of these concept blocks (for example, Hippo–YAP + bioelectricity, and Hippo–YAP + gap junctional communication). The scarcity of the literature jointly addressing all three pillars in the oral cancer context is itself a finding that motivates this review. Foundational and mechanistic studies from outside the oral cancer field, in particular, bioelectric signalling and Hippo regulation, were included as they established principles relevant to the proposed framework. The reference list was further developed by backward and forward citation tracking from key mechanistic papers. Sources were selected based on their relevance to the convergence hypothesis and mechanistic informativeness rather than by predefined systematic inclusion criteria, and the review is accordingly interpretive rather than exhaustive. To make explicit the balance between OSCC/HNSCC-specific evidence and principles from extrapolated fields, all cited sources are categorised by domain in Supplementary Table S1.

## 3. CSC Plasticity and the Attractor Framework in OSCC

The canonical hierarchical model of cancer stem cells (CSCs), in which a genetically fixed apex subpopulation generates non-tumorigenic progeny, cannot account for a growing body of experimental observations: non-CSC populations reliably regenerate CSC fractions after depletion, phenotypically distinct CSC states interconvert, and hybrid epithelial–mesenchymal configurations fit neither pole of the differentiation axis [9,24,25]. In OSCC, single-cell transcriptomic profiling confirms that canonical CSC markers—CD44, ALDH1A1, and CD133—fluctuate across transcriptional continua rather than defining immutable subpopulations [26]. Vipparthi et al. demonstrated that hybrid epithelial–mesenchymal states are the predominant stem-like configuration in oral cancer and correlate with poor clinical outcomes [8]. These observations call for a framework that accommodates reversibility, stochastic state transitions, and phenotypic heterogeneity within genetically identical populations.

The gene regulatory network (GRN) attractor framework, developed by Huang, Ernberg, and Kauffman, provides this framework [10]. In dynamical systems terms, a cell’s phenotype is the product of GRN dynamics that channel cells toward a limited set of stable configurations—attractors—corresponding to distinct, reproducible cell phenotypes. The collection of attractors and the barriers between them constitute the gene regulatory landscape, a formal instantiation of Waddington’s epigenetic landscape metaphor [27]. Cancer arises, in this view, not only through somatic mutation but through deformation of this landscape: mutations lower energy barriers, rendering aberrant attractor states accessible, many of which resemble embryonic or progenitor configurations [6,28]. The critical implication is that the same genome, differently regulated, can generate both normal and malignant phenotypes—establishing the theoretical basis for state-reprogramming rather than purely cytotoxic therapeutic strategies. Intratumoral heterogeneity, long attributed to clonal genetic evolution, is now understood to have a substantial nongenetic component driven by GRN dynamics and stochastic state transitions [29].

The attractor model makes a specific, testable prediction: subpopulations perturbed by sorting or drug treatment should reconstitute the original phenotypic distribution as the system returns to its attractor basin. Li et al. confirmed this bidirectionally in a clonal breast cancer line—both CSC-enriched and non-CSC-enriched fractions regenerated the full phenotypic spectrum within days to weeks—establishing the hallmark behaviour of a dynamical system organised around stable attractors [30]. In OSCC, formal attractor mapping has not yet been performed, but indirect evidence is consistent with this organisation: the CancerSEA atlas reveals discrete co-occurring transcriptional modules (stemness, EMT, proliferation, stress) across OSCC-relevant data [26], and hybrid EMT states, which may represent intermediate basins, exhibit the highest plasticity and worst clinical outcomes [8].

The depth and accessibility of the CSC attractor basin are determined by the architecture of self-reinforcing GRN circuits. In OSCC, the *TAZ–TEAD4–SOX2* positive feedback loop—in which nuclear YAP/TAZ transcriptionally activate *SOX2,* and *SOX2* in turn suppresses Hippo-mediated YAP/TAZ phosphorylation by repressing upstream activators NF2 and WWC1—exemplifies this architecture [14,15]. Co-expression of OCT4 and NANOG with CSC markers in OSCC suggests that embryonic pluripotency GRN modules are reactivated as part of the malignant attractor programme [31], consistent with Huang and Kauffman’s prediction that cancer attractors resemble progenitor states encoded within conserved regulatory circuitry. Taken together, the OSCC CSC state is best understood as a dynamically stabilised GRN attractor maintained by self-reinforcing transcriptional circuits within a deformed regulatory landscape. The central question—what biophysical forces shape this landscape in OSCC—is the subject of the sections that follow.

## 4. Hippo–YAP Signalling as the Gene Regulatory Network Stabiliser of Stemness in OSCC

### 4.1. The Hippo Pathway: Architect and Normal Function

The Hippo pathway operates as a kinase cascade: MST1/2 phosphorylate and activate LATS1/2, which phosphorylate YAP and TAZ, sequestering them in the cytoplasm via 14-3-3 binding and targeting them for proteasomal degradation [32]. When the pathway is inactive, unphosphorylated YAP and TAZ translocate to the nucleus and bind TEAD family transcription factors to drive proliferation, survival, EMT, and stem cell maintenance. Upstream regulation is rich: apicobasal polarity complexes, adherens junctions, and F-actin cytoskeletal tension all modulate LATS1/2 activity, positioning Hippo as an integrator of mechanical, polarity, and intercellular adhesion cues [33]. This mechanosensory function extends to transmembrane voltage gradients, as discussed in Section 4.5, a finding central to the three-pillar convergence hypothesis of this review.

### 4.2. Genomic Evidence of Hippo–YAP Dysregulation in OSCC and HNSCC

The case for Hippo–YAP as a driver of OSCC biology begins at the genomic level. Comprehensive characterisation of HNSCC identified recurrent focal amplification at chromosome 11q encompassing YAP1, placing the primary Hippo effector within one of the most recurrently amplified regions in this cancer type [34]. Pan-cancer TCGA analysis confirmed that 14% of HNSCCs harbour combined YAP1 or TAZ amplifications (mutually exclusive pattern), and elevated YAP/TAZ transcriptional target gene activity (22-gene downstream signature) was independently associated with decreased overall survival [35]. FAT1, encoding a protocadherin that scaffolds the Hippo signalling complex, is among the most frequently mutated genes in HNSCC (29.8%), and its loss-of-function activates YAP1 through failure of MST1 membrane recruitment [36]. Atypical, non-canonical Hippo alterations independent of upstream kinase suppression have additionally been characterised as emerging hallmarks of head and neck cancer biology [12].

Functional evidence comes from a genome-wide CRISPR-Cas9 fitness screen of OSCC cell lines in which YAP1 and TAZ emerged as essential genes: genetic ablation induced apoptosis and substantially reduced viable cell populations, demonstrating a fitness addiction to sustained YAP/TAZ activity [13]. Transcriptional profiling of dependency classes revealed distinct programmes: YAP1-dependent tumours enriched for cell cycle signatures, WWTR1 (TAZ)-dependent tumours enriched for immune and interferon-response signatures. YAP1 was independently identified as a candidate oncogene on the chromosome 11q22 amplicon [37], and its oncogenic credentials are further supported by a systematic review of 13 OSCC studies finding pro-oncogenic direction in 12/13 (92%), with unfavourable direction in all studies examining EMT, migration, invasion, and in vivo tumour growth [38].

### 4.3. YAP as a Driver of Stemness and Malignant Reprogramming in OSCC

YAP functions not merely as a proliferative switch but as a reprogramming factor capable of inducing stem-like transcriptional states in committed epithelial cells. Omori et al. demonstrated using an inducible mouse model that endogenous YAP1 hyperactivation (through Hippo pathway inactivation) in oral epithelium is sufficient to drive the onset and progression of OSCC, establishing YAP1 as a potent oncogenic driver rather than a passive amplification bystander [39]. The most mechanistically resolved evidence for YAP as a CSC attractor-inducing factor comes from Faraji et al., who used spatiotemporally controlled oncogene activation with multiomics in KRT14^+^ oral epithelial progenitors [40]. Constitutively active YAP induced invasive oral carcinoma with high penetrance, and single-cell RNA sequencing identified convergence into a discrete tumour-initiating (TI) cell cluster absent from normal epithelia, enriched for YAP, mTORC1, E2F, MYC, partial EMT, and squamous differentiation signatures. TI cell programmes were conserved in human TCGA-HNSC tumours where above-median YAP and mTORC1 activity independently associated with worse overall and disease-free survival. YAP directly activated NRG1 and AXL (EGFR/HER3 axis driving mTOR signalling) and PDPN (pEMT marker), and mTOR inhibition substantially reduced carcinoma formation in vivo, identifying a YAP→mTOR vulnerability with direct therapeutic implications.

At the histologic and clinical level, Amano et al. performed the first comprehensive IHC characterisation of major Hippo pathway proteins across OED, CIS, and OSCC (*n* = 109): MST1, LATS1, and LATS2 were consistently low across all stages, while YAP1 nuclear expression increased with tumour grade and infiltrative invasion pattern, correlating with EMT markers (E-cadherin loss, vimentin, Slug) and with PRMT1/5 co-expression as an independent predictor of relapse-free survival [41]. YAP and TAZ are essential for basal and squamous cell carcinoma initiation [42], and nuclear YAP consistently correlates with aggressive tumour behaviour, lymph node metastasis, and reduced survival across the OSCC clinical literature. This body of evidence collectively positions nuclear YAP as the convergence output of upstream biophysical dysregulation and as the functional engine of the OSCC CSC attractor state.

### 4.4. The TAZ–TEAD4–SOX2 Axis: Mechanistic Link Between Hippo and Stemness GRNs

The mechanistic bridge between Hippo–YAP pathway activity and the CSC gene regulatory network is most clearly established through the *TAZ–TEAD4–SOX2* transcriptional axis. Li et al. demonstrated that in head and neck squamous cell carcinoma, TAZ directly binds to TEAD4 and the complex occupies the transcriptional promoter of *SOX2*, driving its expression and thereby activating the broader pluripotency-associated GRN that sustains CSC self-renewal [14]. SOX2, a master transcriptional factor of embryonic and adult stem cell identity, is a critical node in the OSCC CSC GRN: its overexpression is sufficient to promote tumour initiation/sphere formation in squamous carcinoma models [43], and its expression correlates with poor prognosis in OSCC patients [44]. The TAZ–TEAD4–SOX2 circuit therefore represents a direct transcriptional mechanism by which Hippo pathway inactivation, signaled by loss of upstream kinase activity or by mechanical/bioelectric cues that prevent LATS1/2-mediated phosphorylation, translates into activation of the stemness GRN.

The feedback architecture of this circuit is equally important. SOX2 has been shown to antagonise the Hippo pathway through direct transcriptional repression of two upstream Hippo activators, NF2 (Merlin) and WWC1 (Kibra), thereby relieving upstream restraint on YAP and reinforcing its nuclear retention [15]. It should be noted that this mechanism was established in osteosarcoma and glioblastoma cell lines; direct evidence in oral epithelial or HNSCC models has not been reported. This creates a double-positive feedback loop—TAZ activates SOX2, and SOX2 in turn stabilises TAZ/YAP nuclear activity—that constitutes precisely the kind of self-reinforcing transcriptional circuit that, in the attractor framework described in Section 3, deepens the CSC attractor basin and raises the energy barrier to exit.

Beyond direct activation of the stemness gene programme through TAZ–TEAD4–SOX2, nuclear YAP additionally drives EMT-associated transcriptional reprogramming in oral epithelium. Xu et al. demonstrated that ECM stiffness-induced Piezo1 activation in oral epithelial cells produces YAP nuclear translocation that upregulates vimentin and downregulates E-cadherin, the canonical EMT transcriptional signature [20]. EMT, particularly in hybrid epithelial–mesenchymal configurations, is closely associated with CSC self-renewal, plasticity, and tumour-initiating capacity in OSCC and oral epithelial cancer, with single-cell transcriptomic studies in OSCC documenting hybrid epithelial–mesenchymal states enriched for CSC markers [8]. The YAP convergence node therefore drives at least two complementary transcriptional outputs that together produce the full CSC phenotype: the TAZ–TEAD4–SOX2 axis activates stemness genes directly, while the YAP–EMT axis produces the phenotypic plasticity characteristics of CSC states. SOX2 functions as a shared transcriptional integrator of both axes, since it has additionally been implicated in driving mesenchymal gene expression while self-amplifying through NF2/WWC1 repression. Although this dual-axis architecture has not been formally tested in OSCC, the convergent activation of stemness and EMT programmes through a shared YAP–SOX2 transcriptional core provides a parsimonious mechanism for the depth and self-reinforcing nature of the OSCC CSC attractor state, and predicts that experimental disruption of either axis alone, without simultaneous disruption of the other, would produce only transient CSC displacement.

Direct support for both arms of this dual SOX2 role has since been reported in OSCC. In CAL27 and SCC15 tongue squamous cell carcinoma cells, transient *SOX2* knockdown reduced the mesenchymal markers vimentin and SNAIL, with a modest increase in E-cadherin, while simultaneously lowering 3D sphere-forming capacity and cell migration and attenuating AKT signalling [45]. These data substantiate, within oral tissue, both stemness-maintaining and EMT/invasion-promoting functions attributed to SOX2 in the convergence model and link the SOX2 node to the PI3K/AKT/mTOR axis discussed in Section 7. The SOX2→NF2/WWC1 repression that closes the self-reinforcing loop, however, remains established in osteosarcoma and glioblastoma; confirming this specific repression in oral cells is identified as a priority in Section 9.1.

Li et al. further showed that TAZ promotes EMT and CSC maintenance in oral cancer through regulation of EMT-associated genes including vimentin and fibronectin, connecting the Hippo–SOX2 axis to the broader phenotypic plasticity programme [44]. The co-amplification of *YAP1* and *SOX2* at chromosomes 11q and 3q, respectively in HNSCC, both within the most recurrently gained chromosomal regions [34], is consistent with a model in which genomic co-amplification reinforces the TAZ–SOX2 positive feedback loop at the gene dosage level [34].

### 4.5. Membrane Potential as an Upstream Regulator of Hippo–YAP: The Mechano-Electric Bridge

A critical and recently published mechanistic insight positions Hippo–YAP not only as a downstream effector of mechanical inputs but as a sensor of transmembrane voltage states, directly connecting the bioelectricity pillar of this review’s hypothesis to the Hippo–YAP pillar at a molecular level. Mukherjee et al. demonstrated in a landmark *Cell* 2026 study that membrane potential mediates the cellular response to mechanical pressure through a mechano-electro-osmotic mechanism with direct consequences for YAP nuclear localisation [16].

In their model, a reduction in tissue biomass density, as occurs during wound healing when cells are mechanically stretched, triggers membrane depolarisation, promotes YAP nuclear translocation (via FAT1-mediated disassembly of the MST1 signalling complex), stimulating cell growth and cycle progression to restore tissue homeostasis. Conversely, high tissue density imposes mechanical compression and membrane hyperpolarisation, which activates MST1, leading to LATS1/2-mediated YAP phosphorylation and cytoplasmic sequestration, thereby suppressing proliferation. This bidirectional, causal connection between membrane potential and Hippo pathway activity established V_mem_ as an upstream regulatory input to the Hippo kinase cascade; not merely a correlate of cell state but an active determinant of YAP nuclear availability. Importantly, these experiments were conducted in kidney (MDCK) and mammary epithelial cell lines (MCF10A, EpH4-Ev, primary HMECs); direct demonstration of this mechanism in oral epithelial or OSCC systems has not yet been reported.

To our knowledge, no study has yet demonstrated the V_mem_ → MST1 → Hippo mechanism directly in oral epithelial or HNSCC cells. The evidence in oral tissue is at present indirect, comprising the Piezo1-mediated mechanotransduction route reported by Xu et al. in oral submucous fibrosis and the established genomic and functional roles of FAT1 and Hippo effectors in OSCC [13]. The bioelectric-to-Hippo connection in oral tissue is therefore proposed here as an extrapolation requiring direct experimental confirmation, not an established fact.

A second feature of the Mukherjee mechanism, underemphasised but critical for the OSCC context, is that bioelectric homeostasis in epithelium operates at two distinct scales. At the single-cell scale, V_mem_ responds to mechanical pressure through the mechano-electro-osmotic coupling described above. At the tissue level, Mukherjee et al. observed that this V_mem_-dependent growth control is calibrated against the achievement of tight barrier function, with growth proceeding until barrier integrity establishes the homeostasis endpoint signal that constrains further proliferation [16]. Tight junctions, through their role in establishing transepithelial potential difference (TEPD), generate the tissue-level bioelectric reference that single-cell V_mem_ signalling drives forward. Saw et al. independently demonstrated that TEPD itself governs epithelial homeostasis through electromechanical coupling [46]. This two-scale architecture has direct implications for the OSF-to-OSCC continuum: OSF is characterised by epithelial atrophy, loss of normal architecture, and disruption of barrier integrity, all of which would compromise the tissue-level TEPD that normally anchors the homeostatic feedback signal. In OSF tissue, oral epithelial cells may therefore experience not only individual V_mem_ dysregulation but also the loss of tissue-level reference that tells them when proliferation should cease—a homeostatic system without a reachable endpoint.

The Mukherjee mechanism additionally couple bioelectric and mechanotransductive signalling at the molecular level. Mukherjee et al. note that membrane potential is well-positioned to regulate any signalling pathway originating at the plasma membrane, and they specifically identify calcium signalling as an “ideal effector” of V_mem_-mediated mechanical transduction, citing the strong voltage-dependence of calcium ion flux. Critically, they observe that flux through key calcium channels including Piezo is voltage-dependent, with Piezo channels themselves shown to be voltage-gated [16]. This intrinsic voltage-sensitivity of Piezo, demonstrated experimentally by Moroni et al., means that V_mem_ state co-regulates Piezo channel calcium influx in response to a given mechanical stimulus [47]. The Piezo1-mediated mechanotransduction route demonstrated by Xu et al. [20] in OSF therefore does not operate independently of cellular V_mem_ state. In OSF/OSCC, where both V_mem_ dysregulation and ECM stiffening occur simultaneously, the bioelectric and mechanotransductive systems are not parallel insults but coupled signalling architectures whose dysregulation compounds at the channel level.

The implications for OSCC carcinogenesis are direct. Ion channel dysregulation, a well-documented feature of cancer cells, alters membrane potential in ways that may render cells unresponsive to the mechanical cues that normally activate the Hippo pathway and suppress YAP [19]. A depolarised membrane state, whether arising from ion channel mutation, altered expression, or disrupted gap junctional coupling, would—by extension of the Mukherjee biomass-density model, though this specific extrapolation has not yet been directly demonstrated in cancer—be expected to favour YAP nuclear retention independently of tissue density cues. In the context of the OSF-to-OSCC continuum, the Piezo1-mediated mechanosensory pathway identified by Xu et al. [20] provides a complementary upstream route—increased ECM stiffness activates Piezo1, driving calcium influx and YAP nuclear translocation, priming the oral epithelium toward a pro-malignant configuration. Together, the bioelectric and mechanotransductive pathways, coupled at Piezo1 voltage-gating and converging at intracellular calcium, suggest that the oral epithelium in betel quid-associated OSCC is subject to convergent biophysical inputs that both favour sustained YAP nuclear activity and CSC attractor stabilisation.

This mechano-electric bridge to Hippo–YAP is the molecular convergence point of the three-pillar hypothesis advanced in this review, and it is developed further in Section 5 and Section 6 in the context of bioelectric signalling and gap junctional communication respectively.

## 5. Bioelectric Signalling as a Higher-Order Regulator of Epithelial Cell Fate and CSC Attractor Stabilisation

Membrane potential (V_mem_) is not merely a metabolic epiphenomenon but an instructive regulator of cell fate and tissue organisation. Binggeli and Weinstein first proposed in 1986 that membrane depolarisation could constitute a general mechanism of growth regulation and cancer formation [17], a prediction supported by decades of subsequent evidence: resting V_mem_ is systematically more hyperpolarised in quiescent or differentiating cells and more depolarised in proliferating cells, and tumour cells across multiple cancer types are consistently more depolarised than their normal tissue counterparts [48]. A recent meta-analysis quantified this signature across 41 cancer cell types, finding mean polarisation differentials of approximately 16 mV in human and 32 mV in rodent cancer cells relative to their normal counterparts [49]. Levin and colleagues demonstrated in developmental biology models that V_mem_ patterns in embryonic tissues encode positional and fate information independently of genetic sequence, and that experimental manipulation of these patterns can redirect tissue-level outcomes including organ identity, regeneration, and tumour suppression [18]. Critically for this review, Saw et al. demonstrated that transepithelial potential difference (TEPD) governs epithelial homeostasis through an electro-osmotic mechanism in polarised epithelia: basal-to-apical TEPD promotes junction formation and uniform morphology, while reversal induces proliferative bud-like structures, establishing that V_mem_ is a tissue-level organiser, not a cell-autonomous property [46]. As introduced in Section 4.5, this two-scale architecture is fundamental to oral epithelial homeostasis: single-cell V_mem_ regulates individual cell-fate decisions through Mukherjee’s mechano-electro-osmotic mechanism, while tissue-level TEPD established by tight epithelial barrier functions provides the homeostatic endpoint signal that single-cell V_mem_ signalling drives toward [16,46]. Both scales are dysregulated in the OSF-to-OSCC continuum: individual oral epithelial cells experience V_mem_ perturbation from arecoline-induced ROS and altered ion channel function, while OSF tissue architecture compromises tight junction integrity and therefore the TEPD-based homeostatic reference.

The mechanistic bridge between V_mem_ and Hippo–YAP signalling was established by Mukherjee et al., who demonstrated in a non-oral epithelial system that changes in cellular biomass density alter V_mem_ through an electro-osmotic mechanism, and that this V_mem_ shift in turn regulates MST1 kinase activity: hyperpolarisation activates MST1 → LATS1/2 → YAP phosphorylation and cytoplasmic retention, while depolarisation reduces LATS activity and promotes YAP nuclear translocation [16]. Critically, Mukherjee et al. demonstrated that this V_mem_–Hippo signalling axis is FAT1-dependent: in MCF10A cells, FAT1 knockdown abolished the YAP localisation response to valinomycin-induced depolarisation, identifying FAT1 not as a parallel co-regulator of Hippo activity but as a required scaffold for MST1 membrane recruitment in response to V_mem_ changes [16]. The implication for HNSCC is direct: in the 29.8% of HNSCC harbouring FAT1 loss-of-function mutations [36], the bioelectric pillar of the convergence framework is structurally decoupled from the Hippo pillar at the molecular level. V_mem_ changes, whether arising from arecoline-induced ROS, ECM stiffness perturbation, or ion channel dysregulation, cannot reach LATS1/2 to restrain YAP nuclear retention, because the FAT1-dependent MST1 membrane recruitment step is missing. FAT1 mutation therefore does more than amplify YAP activation; it produces a state in which YAP nuclear retention becomes uncoupled from the homeostatic V_mem_ feedback that would normally constrain it. This extrapolation from the experimental knockdown phenotype to FAT1-mutant tumours, while biologically plausible, has not been tested in OSCC. This structural difference implies that the convergence model is not uniform across patients—the upstream bioelectric and gap junctional inputs can modulate Hippo activitiy only where FAT1 is intact, whereas in FAT1-altered tumours, nuclear YAP is sustained more directly through loss of the FAT1–MST1 restraint. The therapeutic consequences of this division are developed in Section 8.4.

As developed in Section 4.5, the bioelectric and mechanotransductive routes to YAP nuclear retention are coupled rather than parallel. Mukherjee et al. observe that V_mem_ is well-positioned to regulate any signalling pathway originating at the plasma membrane, identifying calcium signalling as an “ideal effector” of V_mem_-mediated mechanical transduction given the strong voltage-dependence of calcium ion influx [16]. Critically, Piezo channels, the mechanosensors that drive YAP nuclear translocation in OSF [20], are themselves voltage-gated, with channel inactivation kinetics demonstrated experimentally to depend on cellular membrane potential [47]. This intrinsic voltage-sensitivity of Piezo1 means that the calcium influx response to a given mechanical stimulus depends on the cell’s V_mem_ state. In oral epithelium subjected simultaneously to ECM stiffening and V_mem_ dysregulation, the prevailing condition in OSF-to-OSCC progression, the two perturbations integrate at the Piezo1 channel itself rather than acting as independent insults. Beyond Piezo1, Mukherjee et al. additionally demonstrated that V_mem_ modulates mitogen-activated protein kinase (MAPK) signalling, possibly downstream of recently characterised V_mem_–K-Ras coupling [16], suggesting that bioelectric dysregulation in OSCC may have regulatory consequences beyond Hippo–YAP that warrant investigation. Independent evidence from a non-epithelial system further supports a direct ion channel to Hippo link; Dupuy et al. demonstrated that transcriptional regulation of the potassium channel KCNA2 (Kv1.2) controls YAP/Hippo signalling and cell proliferation, establishing that specific ion channel activity can directly modulate Hippo pathway output [50]. Together, these findings support a multi-input bioelectric–Hippo axis in which membrane depolarisation, whether arising from altered ion channel expression, disrupted GJIC, or ECM-driven mechanosensory signals, converges on reduced LATS1/2 activity and sustained YAP nuclear retention through FAT1-dependent and -independent routes.

In the attractor framework developed in Section 3, sustained V_mem_ depolarisation is proposed to function as a landscape-deforming force in OSCC: by persistently reducing LATS1/2-mediated YAP phosphorylation independently of upstream oncogenic signals, it deepens the CSC attractor basin and raises the energy barrier to differentiation. No published study has simultaneously measured V_mem_ profiles, ion channel transcriptomes, and CSC marker expression within the same OSCC model—this constitutes the primary experimental gap motivating the bioelectric axis of the convergence framework. In the betel quid-associated OSCC context, the OSF tissue environment engages multiple V_mem_-perturbing inputs simultaneously: arecoline-induced mitochondrial ROS alter ion channel function and shift V_mem_ toward depolarisation, providing a chemical carcinogen-driven bioelectric dysregulation route; ECM stiffening drives Piezo1-mediated calcium influx, with the magnitude of this response itself dependent on V_mem_ state through Piezo voltage-gating; and FAT1 loss-of-function in approximately 30% of HNSCC structurally decouples the bioelectric input from Hippo regulation. The mechanistic implication is that betel quid is uniquely damaging not because it engages multiple pathways additively but because the bioelectric and mechanotransductive systems normally provide redundant fail-safe that fail together; arecoline disrupts the V_mem_-mediated route, OSF disrupts the mechanotransductive route, and the two routes are coupled at Piezo1 voltage-gating such that disruption of one amplifies dysregulation of the other. Whether this proposed multiplicative coupling produces measurably greater YAP nuclear retention than would be predicted by additive insults is directly testable and constitutes a tractable experimental question within the convergence framework.

## 6. Gap Junctional Intercellular Communication (GJIC), Connexin Dysregulation, and the Fragmentation of Epithelial Electric Coherence in OSCC

### 6.1. GJIC as the Integrator of Individual Cell Bioelectric States

Gap junctions are intercellular channels formed by connexin hemichannels that enable the direct cytoplasmic exchange of ions, second messengers (Ca^2+^, cAMP, IP_3_), and metabolites up to ~1 kDa between adjacent cells, constituting the principal mechanism by which individual cell bioelectric states are integrated into tissue-level electrical fields [51]. In oral epithelium, Cx43 (GJA1) and Cx26 (GJB2) are the predominant isoforms, with expression tightly coupled to keratinocyte differentiation state, positioning connexins as markers and mediators of epithelial cell fate [52]. Cx43 function is regulated beyond transcription: phosphorylation by Src and EGFR—both constitutively activated in OSCC—reduces channel conductance and accelerates protein degradation, providing a post-translational route to GJIC disruption that is independent of connexin gene expression [51,53]. In the majority of epithelial cancers, GJIC is substantially reduced relative to normal tissue through a combination of transcriptional downregulation, promoter hypermethylation of GJA1, oncogenic kinase-mediated channel inactivation, and redistribution of connexin from the plasma membrane to intracellular compartments [54,55]. This reduction correlates with tumour grade and invasive capacity across multiple cancer types. The functional consequence most relevant to this review is the loss of collective bioelectric error-correction: when GJIC is intact, a cell entering an aberrant bioelectric state receives corrective signals from its neighbours through shared ion flux; when GJIC is disrupted, local epithelial clusters can sustain aberrant V_mem_ and attractor states independently of tissue-level cues. It should be noted that connexin biology in cancer is context-dependent: in some settings, connexins facilitate tumour progression through metabolic coupling with cancer-associated fibroblasts or metastatic niche formation, underscoring the need for OSCC-specific rather than extrapolated evidence [54].

### 6.2. Connexin Expression and GJIC in OSCC: Current Evidence and Critical Gaps

Direct OSCC evidence for connexin dysregulation is limited in scope but consistent in direction. Immunohistochemical studies confirm reduced or redistributed Cx43 relative to normal oral mucosa [54]. The most detailed OSCC IHC study found that functional GJIC loss reflects cytoplasmic sequestration of Cx43 rather than transcriptional silencing—cytoplasmic Cx43 was significantly upregulated while membrane Cx43 was not reduced in aggregate [52]. Critically, high membrane Cx43 was the only independent predictor of shortened overall survival (*p* = 0.0088), suggesting a context-dependent tumour-promoter role for membrane-localised Cx43 in established OSCC that underscores why expression levels alone, without subcellular localisation data, are insufficient to infer GJIC status. This finding is corroborated in a 90-patient HNSCC tissue microarray: Cx43-negative tumours had median disease-specific survival of 5 months versus 15 months in Cx43-positive tumours (HR = 0.509; *p* = 0.004), though oral cavity primaries comprised only 5% of that cohort [56]. For the betel quid-specific OSF-to-OSCC transition, Mugundan et al. performed IHC for Cx43 across normal mucosa (n = 6), OSF (n = 14), oral epithelial dysplasia (n = 12), and OSCC-with-OSF history (n = 7): Cx43 showed a stepwise reduction across groups (*p* = 0.033), with OSF retaining moderate suprabasal expression, OED showing progressive loss, and OSCC-with-OSF showing near-complete loss in infiltrating tumour islands (OSF vs. OSCC-with-OSF: *p* = 0.014) [57]. Separately, Gutierrez-Camacho et al. reported that Cx43 expression serves as a biomarker discriminating OSCC from normal oral mucosa, with additional associations between Cx43 loss and HPV16/18 status [58]. This stepwise loss indicates that GJIC disruption occurs during, rather than prior to, malignant transformation from the OSF precursor field, consistent with a causal role, though causality has not been directly demonstrated. Functional GJIC also governs stem cell fate in progenitor contexts: Ho et al. demonstrated that gap junction-mediated calcium signalling controls blood progenitor cell fate decisions in haematopoiesis, establishing GJIC as an active determinant of stem cell state transitions [59], though the downstream effector in that system was CaMKII–JAK–STAT rather than CaMKII–MST1–Hippo, so the mechanistic analogy remains inferential. The critical gap is that no published study has simultaneously characterised connexin expression, GJIC functional capacity, and CSC marker expression within the same OSCC model.

### 6.3. The Proposed Mechanism: GJIC Fragmentation and Local CSC Attractor Emergence

In the attractor framework described in Section 3, the CSC state is a stable but locally accessible configuration on the gene regulatory network, a basin that cells normally cannot enter easily because the surrounding regulatory landscape maintains differentiated attractor states. The bioelectric model developed in Section 5 proposed that tissue-level V_mem_ dysregulation deforms this landscape, making the CSC basin more accessible. GJIC fragmentation extends this model by removing the collective error-correction mechanism that normally prevents local cells from entering aberrant attractor states independently of the tissue collective.

The proposed mechanism operates as follows. In normal oral epithelium, GJIC maintains synchronised bioelectric states across the epithelial sheet. A cell that transiently depolarises, perhaps in response to a local mechanical perturbation or a burst of ROS from carcinogen exposure, is rapidly corrected by ion flow from hyperpolarised neighbours through gap junctions, restoring it to the tissue-appropriate V_mem_ state and maintaining Hippo pathway activity. This collective buffering capacity means that individual cells rarely sustain the prolonged depolarisation needed to maintain YAP in the nucleus long enough to initiate the TAZ–SOX2 positive feedback loop.

When GJIC is fragmented, whether by connexin downregulation, Cx43 phosphorylation by oncogenic kinases, or fibrotic ECM remodeling, this buffering capacity is lost. Local clusters of cells become electrically isolated from the surrounding epithelium, allowing transiently depolarised cells to sustain their altered V_mem_ state without correction.

Computational models of coupled bioelectric-transcriptional networks formalise this principle. Cervera et al. demonstrated through simulations of multicellular ensembles that intercellular junction conductance is the parameter determining whether individual-cell V_mem_ perturbations are buffered through a community effect of neighbouring cells or whether they persist as stable local regionalisation [60]. In their model, strong intercellular coupling produces a synchronised polarisation state across the multicellular system, with individual aberrant cells rapidly corrected toward the tissue-level V_mem_; weak coupling permits sustained local heterogeneity, with depolarised regions persisting independently of neighbouring cells and feeding back into distinct local transcriptional programmes. This computational framework provides theoretical grounding for the proposed mechanism: GJIC fragmentation in OSCC is expected to convert single-cell bioelectric noise, which would otherwise be transient and rapidly corrected, into stable multicellular regionalisations of aberrant V_mem_ state, each capable of sustaining local YAP nuclear retention long enough to engage to the TAZ–SOX2 self-reinforcing circuit. Although the Cervera et al. [59] model was developed for embryogenesis and regeneration contexts rather than carcinogenesis, the underlying principle—that GJIC determines whether cells experience their bioelectric environment collectively or autonomously—applies directly to the OSF-to-OSCC continuum, where progressive Cx43 downregulation and fibrotic disruption of gap junction plaques would predict precisely the kind of coupling collapse that converts buffered bioelectric perturbation into sustained local CSC-permissive states.

Sustained depolarisation, acting through the Mukherjee mechano-electro-osmotic mechanism, maintains YAP nuclear retention in these isolated clusters. If this state persists long enough to initiate the TAZ–SOX2 feedback circuit, the cluster transitions into the CSC attractor basin, a self-stabilising configuration that no longer requires the initiating bioelectric stimulus.

This mechanism predicts that GJIC disruption is not merely correlated with CSC emergence but is causally upstream of it, a prediction that is directly testable by restoring GJIC pharmacologically (e.g., with connexin-enhancing agents such as danegaptide or rotigaptide) and assessing whether CSC frequency is reduced even in the presence of ongoing carcinogenic exposure.

### 6.4. GJIC, Hippo–YAP, and the Convergence Point

The relationship between GJIC and Hippo–YAP signalling has not been studied directly in OSCC, but indirect evidence from adjacent systems supports a mechanistic connection. Cx43 interacts physically with ZO-1 [61], a tight junction scaffolding protein that also regulates LATS1/2 activity through its association with the Hippo pathway component PTPN14 [62]. Disruption of the Cx43–ZO-1 interaction, which occurs downstream of Src-mediated Cx43 phosphorylation, itself activated by oncogenic signalling, may therefore reduce LATS1/2 activity and favour YAP nuclear retention through a connexin-proximal scaffolding mechanism, independent of V_mem_ effects.

Furthermore, intracellular calcium, the primary second messenger propagated through gap junctions during collective mechanosensing, is a known regulator of MST1/2 kinase activity. Calcium/calmodulin-dependent protein kinase II (CaMKII) has been shown to phosphorylate and activate MST1 in the context of Piezo1-mediated mechanosensing in macrophages, linking calcium influx to Hippo pathway kinase activation, as reviewed in [63]. By analogy, in an epithelium with intact GJIC, it is plausible that a mechanical stimulus induces a calcium wave that activates CaMKII → MST1 → LATS1/2 → YAP phosphorylation across the tissue collective—a homeostatic response that suppresses proliferation in response to crowding. When GJIC is disrupted, calcium wave propagation would be blocked, CaMKII–MST1 activation would not spread, and YAP nuclear retention could persist in isolated cell clusters even under conditions of normal tissue density. This mechanistic inference, though not yet demonstrated in an epithelial or OSCC context, provides a plausible direct biochemical link between GJIC disruption and Hippo pathway inactivation that is distinct from, and potentially additive to, the V_mem_-mediated mechanism described in Section 4.

Together, the V_mem_-mediated and proposed calcium wave-mediated mechanisms suggest that GJIC fragmentation acts on the Hippo–YAP convergence node through at least two parallel routes, making GJIC disruption a particularly potent contributor to CSC attractor stabilisation, and a correspondingly important therapeutic target.

### 6.5. The Knowledge Gap and Its Research Implications

The evidence presented in this section is explicit about its limitations. The connexin–OSCC CSC connection is built from general principles of connexin biology and cancer, limited OSCC-specific expression data, a single proof-of-concept study in haematopoietic progenitors, and mechanistic inference from connexin–Hippo pathway molecular interactions. No primary study directly tests the causal relationship between GJIC and CSC state in OSCC.

The gap is, however, precisely what makes it scientifically valuable. Unlike the bioelectricity gap, where the mechanistic framework exists but OSCC-specific application is absent, the GJIC–CSC gap in OSCC represents an almost entirely uncharted territory with clear biological plausibility, established adjacent evidence, and tractable experimental approaches. Characterising connexin expression across OSCC CSC subpopulations, testing whether GJIC restoration reduces CSC frequency in OSCC cell lines and patient-derived organoids, and examining whether connexin–Hippo pathway interaction is altered in OSCC, these are experiments that are feasible with existing tools and that would directly test a novel hypothesis.

Specific experimental approaches are outlined in Section 9.1.

## 7. The Convergence Model in the Context of Other CSC-Regulating Pathways in OSCC

The canonical pathways governing CSC maintenance and plasticity in OSCC—Wnt/β-catenin, Notch, Hedgehog, TGF-β, and PI3K/AKT/mTOR—have been reviewed in detail elsewhere, including our own comprehensive survey of the experimental CSC literature in OSCC [4]. The framework developed here is not intended to displace these pathways but to identify a biophysical convergence point that operates alongside and frequently intersects them; a complete account of the CSC state must situate the Hippo–YAP node within this broader network.

Several of these pathways are mechanically entangled with the convergence node rather than parallel to it. YAP/TAZ participate in the β-catenin destruction complex, coupling Hippo activity to Wnt output and allowing the two effectors to co-regulate overlapping stemness targets [64,65]; Notch and Hedgehog/GLI signalling likewise crosstalk with YAP through shared transcriptional programmes [66]. Two pathways are especially pertinent to the betel quid/OSF context: TGF-β, a principal driver of the fibrotic ECM modelling and EMT that characterise OSF, requires YAP/TAZ as co-effectors of SMAD-driven EMT, placing it upstream of and convergent with the Hippo node during malignant transformation [67,68]; and PI3K/AKT/mTOR, frequently activated in HNSCC functions [69,70] in parts as an effector arm of the convergence node, since YAP drives NRG1/AXL–mTORC1 signalling in oral tumour-initiating cells (Section 4.3) [40]. m-TOR-directed therapy, therefore, targets a downstream output of nuclear YAP rather than an independent input.

Positioned against this landscape, the present framework does not claim that Hippo–YAP is the dominant CSC pathway in OSCC; it proposes that biophysical inputs—membrane voltage and gap junctional coupling—feed a convergence node that is itself embedded in, and reinforced by, this wider stemness network. Because several canonical pathways channel through that node (Wnt–YAP, TGF–β–YAP, mTOR downstream of YAP), their intersection is consistent with the convergence logic rather than contradictory to it and clarifies why convergence-directed combination strategies (Section 8.3) may need to account for these parallel inputs.

## 8. Convergence, Therapeutic Implications, and the Case for CSC State Reprogramming in OSCC

### 8.1. The Convergence Point: Nuclear YAP as the Common Regulatory Output

The preceding sections have established three mechanistically interconnected lines of evidence. Section 4 demonstrated that Hippo–YAP dysregulation is genomically frequent and functionally essential in OSCC, and that the TAZ–TEAD4–SOX2 positive feedback loop stabilises the CSC transcriptional state as a self-reinforcing attractor. Section 5 established that V_mem_ dysregulation, through the mechano-electro-osmotic mechanism identified by [16], is an upstream determinant of YAP nuclear localisation, linking the bioelectric state of the oral epithelium to Hippo pathway activity. Section 6 proposed that GJIC fragmentation amplifies this through two parallel routes: loss of collective V_mem_ buffering and interruption of the proposed Ca^2+^ wave-mediated CaMKII–MST1–LATS1/2 activation. These three systems converge on a single regulatory output: nuclear YAP. When all three are simultaneously dysregulated—Hippo pathway inactivated, membrane depolarised, GJIC fragmented—nuclear YAP persists without correction, activates the TAZ–TEAD4–SOX2 GRN, and drives the oral epithelial progenitor into a stable CSC attractor state. The attractor is then self-sustaining: nuclear YAP activates *SOX2*, *SOX2* represses NF2 and WWC1 (relieving upstream Hippo activation), reduced LATS1/2 activity sustains YAP nuclear retention, and the circuit runs independently of further external stimulus.

**Figure 2:**
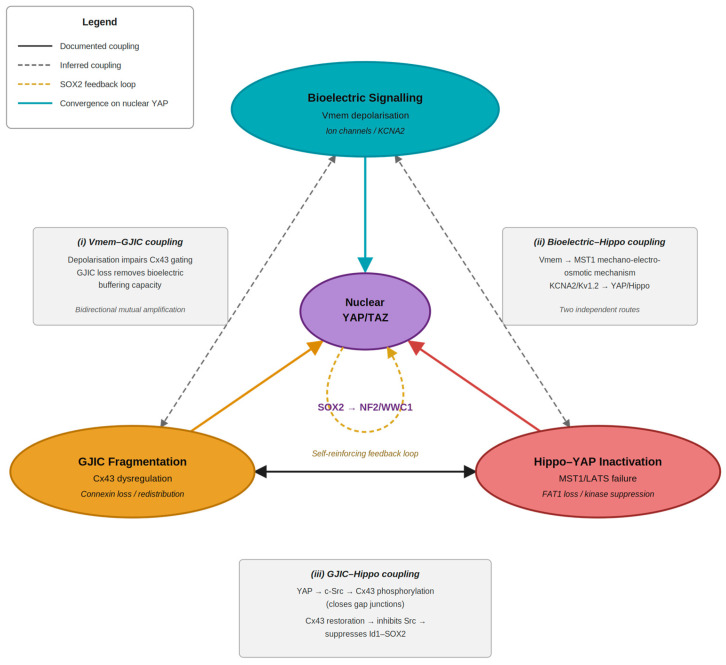
The mutually reinforcing convergence triangle in OSCC CSC attractor stabilisation. The three biophysical pillars—bioelectric signalling (V_mem_ depolarisation, teal), GJIC fragmentation (Cx43, amber), and Hippo–YAP inactivation (MST1/LATS, coral)—are represented as vertices of a triangle, with bidirectional coupling arrows on each edge indicating mutual regulatory relationships. Nuclear YAP (purple) sits at the centre as the convergence node. Solid arrows indicate documented mechanistic coupling; dashed arrows indicate partially inferred or cross-system coupling. Edge annotations cite key mechanistic evidence: (**i**) V_mem_ depolarisation impairs Cx43 hemichannel gating and GJIC conductance, while GJIC loss removes tissue-level bioelectric buffering (bidirectional V_mem_–GJIC edge); (**ii**) KCNA2/Kv1.2 channel activity drives Hippo/YAP signalling and controls nuclear YAP localisation [50], while the V_mem_–MST1 mechano-electro-osmotic mechanism provides a second independent route from the bioelectric pillar to the Hippo pillar [16] (bidirectional bioelectric–Hippo edge); (**iii**) nuclear YAP drives c-Src activation, which phosphorylates Cx43 at Tyr247 and Tyr265, preventing membrane localisation and closing gap junctions (YAP → Src → GJIC route), while Cx43 restoration inhibits c-Src and suppresses Id1–SOX2, reversing the CSC phenotype (Cx43 → Src → YAP route) [71] (bidirectional Hippo–GJIC edge). Three inward spokes represent the common convergence of all three pillars onto nuclear YAP. The gold dashed arc denotes the SOX2 → NF2/WWC1 self-reinforcing feedback loop (SOX2 represses upstream Hippo activators NF2 and WWC1, not LATS2 directly; mechanism established in osteosarcoma/GBM). The figure illustrates that dysregulation of any one pillar amplifies dysregulation of the others, explaining why single-pathway targeting is predicted to be insufficient for durable CSC attractor displacement.

A critical structural question for the convergence model is whether the three pillars are independent parallel inputs to nuclear YAP or a mutually reinforcing triangle in which dysregulation of any one amplifies the others. The available evidence supports the latter. First, nuclear YAP drives c-Src kinase activation downstream, and activated Src phosphorylates Cx43 at Tyr247/Tyr265, preventing membrane localisation and closing gap junctions [53]; conversely, Cx43 restoration in glioma stem cells inhibits c-Src, suppresses Id1 and SOX2, and reverses the CSC phenotype [71], making the Cx43–Src–YAP interaction a self-reinforcing loop. The three nodes of this chain (Cx43 reduced in OSCC, c-Src constitutively activated, *SOX2*-driven self-renewal defining the CSC state) are each independently documented in OSCC, though their causal connectivity in oral squamous epithelium has not been directly tested. Second, V_mem_ dysregulation impairs GJIC directly through connexin hemichannel gating, while GJIC loss prevents tissue-level electrical buffering that normally corrects aberrant single-cell V_mem_. This mutual coupling means that dysregulation of any one pillar tends to amplify dysregulation of the others, explaining why single-pathway targeting is predicted to produce incomplete and transient CSC attractor displacement, and why targeting the convergence node, nuclear YAP, is the most rational intervention point (Figure 2).

### 8.2. Reprogramming Versus Elimination: The Attractor Framework as Therapeutic Rationale

Conventional CSC-targeting strategies aim at elimination of marker-positive stem-like cells. The attractor framework reveals the fundamental limitation of this approach: if CSCs represent dynamic attractor states rather than a fixed subpopulation, elimination will be followed by regeneration as surviving non-CSC cells stochastically re-enter the attractor basin—precisely the phenotypic equilibrium reconstitution demonstrated by Li et al. [30]. Selective cytotoxic pressure may additionally deepen the attractor basin by eliminating non-CSC cells, enriching for therapy resistance. The attractor framework argues instead for deforming the landscape itself: raising the energy barrier around the CSC basin and simultaneously lowering barriers toward differentiated attractors. This reprogramming logic—targeting the regulatory landscape rather than any particular cell population—has proof-of-principle support in multiple cancer systems [72,73] and is the conceptual foundation for the therapeutic strategies discussed below.

### 8.3. Therapeutic Implications: Targeting Nuclear YAP and Its Upstream Regulators

The most direct therapeutic approach is inhibition of the YAP–TEAD transcriptional complex. Verteporfin and CA3 suppress YAP-driven EMT, migration, and xenograft growth in OSCC preclinical models [38]; in an arecoline-induced BALB/c mouse model of OSF, verteporfin treatment specifically suppressed YAP-driven endothelial–mesenchymal transition and reduced submucosal collagen accumulation, providing direct in vivo proof-of-concept for YAP–TAD inhibition as an OSF-stage chemoprevention strategy [23]. TEAD inhibitors IK-930 (NCT05228021) and VT3989 (NCT04665206) are in Phase I/II trials across Hippo-altered solid tumours; VT104, a related compound, significantly reduced clonogenic growth of YAP-activated oral epithelial progenitors in vitro [40], and Sato et al. identified FAT1-altered HNSCC as a potentially enriched-response population [36]. Downstream of YAP, Faraji et al. [40] established that YAP transcriptionally activates NRG1 and AXL, driving mTORC1 signalling, and that rapamycin markedly reduced carcinoma formation in vivo in YAP-activated oral progenitors, identifying TEAD inhibitor + mTOR inhibitor combination as mechanistically motivated (caveat: HPV-driven mouse model; betel quid-specific validation needed). Faraji et al. [40] additionally identified YAP-activated TI cells as the source of CXCL1/CXCL2 that recruit granulocytic MDSCs supplying MMP8/MMP9 collagenases for basement membrane invasion; both G-MDSC depletion (anti-LY6G) and CXCR1/2 inhibition (ladarixin) significantly reduced carcinoma incidence in vivo, motivating a second combination: TEAD inhibitor + CXCR1/2 blockade targeting cell-autonomous CSC programme and non-cell-autonomous invasion in parallel.

The bioelectric and GJIC pillars imply upstream reprogramming strategies distinct from direct YAP targeting. Pharmacologic hyperpolarisation using K_v_-channel openers (retigabine, minoxidil) is predicted, via the Mukherjee V_mem_–MST1 mechanism, to activate LATS1/2 → YAP phosphorylation and reduce CSC frequency in OSCC models—an experiment not yet performed. For GJIC restoration, danegaptide and rotigaptide (connexin-stabilising peptidomimetics developed for cardiac indications) would be predicted to restore Ca^2+^ wave propagation, re-engage CaMKII–MST1 activation in response to cell density cues, and reduce nuclear YAP independently of direct TEAD inhibition [51]. Neither class has been tested in OSCC CSC models; both are tractable with available tools. miRNA-based strategies targeting YAP post-transcriptionally (miR-27a-3p, miR-381-3p, circHIPK3) have shown preclinical activity but face delivery challenges [38]. miR-27a-3p, in particular, was originally identified by Zeng et al. as a direct post-transcriptional suppressor of YAP1 in OSCC cell lines, with restoration of miR-27-3p reducing the YAP1–OCT4 interaction, downregulating SOX2 and EMT transcription factors and impairing invasive capacity, providing OSCC-specific primary evidence for the miRNA–YAP axis as a tractable therapeutic node within the convergence framework [74]. Because several convergence-directed modalities, notably nucleic-acid agents such as YAP-targeting miRNAs and locally acting connexin or ion channel modulators, face bioavailability and tumour-selectivity constraints, nanoparticle- and nanomaterial-based delivery systems represent a relevant avenue for improving their stability, targeting, and clinical translatability [75].

**Figure 3:**
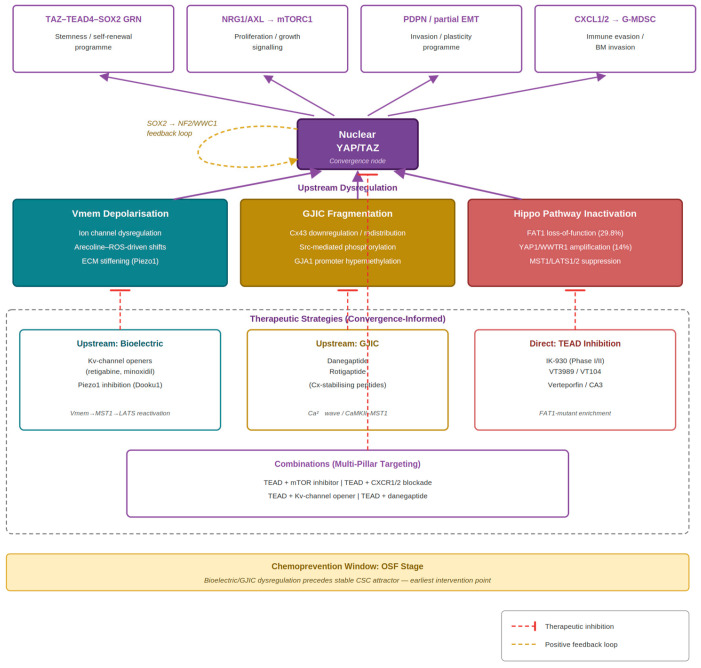
Therapeutic targeting of the three-pillar convergence architecture in OSCC. Three upstream dysregulatory systems (left: Vmem depolarisation, teal; GJIC fragmentation, amber; Hippo pathway inactivation, coral) converge on nuclear YAP/TAZ (centre), which drives four CSC attractor outputs (right: *TAZ–TEAD4–SOX2* GRN, NRG1/AXL → mTORC1 signalling, PDPN/partial EMT, and CXCL1/2 → G-MDSC recruitment). The SOX2 → NF2/WWC1 positive feedback loop (gold dashed arc) reinforces YAP nuclear retention. Purple arrows indicate convergence onto the central node and its downstream transcriptional outputs; red dashed lines indicate therapeutic inhibition (see in-figure legend). Bottom panel: convergence-informed therapeutic strategies spanning direct TEAD inhibition [35] (clinical-stage compounds), mechanistically motivated combinations, and upstream bioelectric and GJIC reprogramming approaches. The OSF chemoprevention window (yellow) identifies the earliest clinically detectable intervention point, where bioelectric and GJIC dysregulation precedes stable CSC attractor formation.

If the three pillars are organised as a convergence architecture instead of as parallel inputs (Section 8.1), single-system targeting is predicted to be insufficient for durable CSC attractor displacement as the remaining stabilising inputs would continue to reinforce nuclear YAP retention. This prediction is therefore conditional on the convergence architecture itself, which remains to be tested (Section 9.1). Combination strategies that simultaneously address multiple inputs—TEAD + mTOR, TEAD + CXCR1/2, K_v_-channel opener + TEAD, or danegaptide + TEAD—are each mechanistically motivated and directly testable in OSCC organoids with CSC frequency and post-withdrawal recurrence as primary endpoints; a finding of non-additive, synergistic benefit from such combinations would itself provide functional support for the convergence architecture, whereas merely additive effects would be equally consistent with parallel regulation. The field cancerization dimension of OSCC pathogenesis further argues for the chemopreventive application of these strategies: the OSF stage, where bioelectric dysregulation, GJIC fragmentation, and Piezo1-mediated YAP activation are each mechanistically plausible but not yet locked into a stable CSC attractor basin, represents a clinically identifiable intervention window with no current pharmacologic equivalent. Bioelectric and GJIC biomarkers that predict malignant conversion in OSF patients would constitute a clinically meaningful output of the mechanistic framework developed here (Figure 3).

### 8.4. Clinical Relevance: Biomarkers, Risk Stratification, and the Prevention Window

Although the convergence framework is mechanistic in construction, its most immediate translational value lies in three clinically actionable directions. First, because the convergence operates substantially at the post-translational level, its readouts are protein-localisation and channel-function assays already routine in diagnostic pathology. Nuclear versus cytoplasmic YAP/TAZ, membrane versus cytoplasmic Cx43 [52], and SOX2 positivity together index the activity of the proposed convergence node and could be combined into a single immunohistochemical panel. Applied to oral potentially malignant disorders, and to OSF, in particular, such a panel offers a candidate biomarker of malignant-conversion risk, distinguishing lesions that are bioelectrically and junctionally “primed” from those that are not, addressing an unmet need, since current OSF surveillance relies on dysplasia grading, a weak predictor of progression [76,77,78,79]. Consistent with such a primed field, SOX2 has been found upregulated in the close resection margins of OSCC relative to healthy mucosa, where its expression correlates with both tumour size and lymph-node compromise [45]. Direct human evidence is that a stemness-associated programme is already active in marginal tissue, supporting the biologically altered field that this prevention argument presupposes.

Second, the framework defines a molecularly selected population for convergence-directed therapy. FAT1 loss-of-function (~30% of HNSCC) and YAP1/WWTR1 amplification (11q/3q) are recurrent, routinely assayable events that the model predicts should sensitise tumours to YAP–TEAD-directed therapy; FAT1-altered HNSCC has already been nominated as an enriched-response population for TEAD inhibitors [36]. Coupled with the protein-level panel above, these markers could support biomarker-stratified enrolment in early-phase trials of clinical-stage TEAD inhibitors (IK-930, VT3989) and their combinations. This stratification cuts in both directions. Because the upstream bioelectric and gap junctional interventions considered here act on Hippo through the FAT1-dependent V_mem_ → MST1 → LATS1/2 route, they are predicted to be effective principally in FAT1-intact tumours; in FAT1-altered tumours, where that route is uncoupled, direct YAP–TEAD inhibition is the more logical intervention. The model therefore predicts a FAT1-status-dependent division of therapeutic strategy (upstream biophysical modulation for FAT1-intact disease and direct nuclear-YAP/TEAD targeting for FAT1-altered disease)—a concrete, assayable prediction since FAT1 status is a routinely obtainable genomic readout.

Third, and most distinctively, the framework identifies the OSF stage as a clinically detectable chemoprevention window in a population of approximately 600 million betel quid users for whom approved targeted antifibrotic or molecularly guided preventive strategies are still lacking [80,81,82,83]. Because several of the implicated upstream agents are already approved or in clinical development for non-oncological indications, for example, K_v_-channel openers (retigabine, minoxidil), connexin-stabilising peptidomimetics (danegaptide, rotigaptide), and Piezo1 inhibitors, the convergence logic is unusually amenable to repurposing at the prevention stage, before the CSC attractor is established. A computational companion to this review (manuscript in preparation) formalises the convergence architecture as a dynamical (Boolean and reduced-ODE) model and uses it to rank these single-agent and combination strategies by predicted durability; in that analysis, a non-additive, synergistic response to specific combinations emerges as the signature that would distinguish a true convergence architecture from parallel regulation, and prioritises these combinations for the experimental agenda in Section 9.1.

## 9. Research Agenda, Future Directions, and Conclusions

### 9.1. From Framework to Evidence: A Prioritised Research Agenda

The convergence framework developed here generates five prioritised experimental questions, each tractable with existing tools and OSCC-relevant model systems. First, formal attractor landscape mapping in OSCC—using single-cell RNA sequencing, GRN reconstruction (SCENIC, Arboreto), and phenotypic reconstitution experiments—is needed to confirm that the CSC state constitutes a discrete transcriptional attractor basin. The Faraji et al. scRNA-seq datasets (GEO: GSE276778–GSE276783) provide a genetically defined oral epithelial CSC entry system for cross-species attractor alignment. Second, electrophysiological characterisation of resting V_mem_ in OSCC CSC versus non-CSC subpopulations, combined with pharmacologic hyperpolarisation using K_v_-channel openers, would directly test whether V_mem_ manipulation alters YAP nuclear localisation and CSC frequency in an OSCC-specific system. Third, connexin isoform profiling and functional GJIC measurement (FRAP assay) across OSCC CSC and non-CSC subpopulations, followed by pharmacologic GJIC restoration using danegaptide, would test whether GJIC re-engagement reduces CSC frequency through Hippo kinase reactivation without requiring direct YAP inhibition. Fourth, characterisation of Piezo1–YAP axis activation in OSF-derived primary epithelial cells and patient organoids on stiffness-graded matrices would test whether Piezo1 inhibition (Dooku1) prevents CSC state enrichment at the OSF stage, a potential chemoprevention window. Fifth, systematic preclinical combination testing—pairing TEAD inhibition with mTOR inhibition, CXCR1/2 blockade, K_v_-channel openers, or GJIC restoration—in OSCC cell lines and patient-derived organoids, with CSC frequency and post-withdrawal recurrence as primary endpoints, would test the core prediction of the convergence model: that multi-system perturbation produces the non-additive, durable CSC attractor displacement that single-pathway targeting cannot achieve. Collectively, these experiments would transform the framework from synthesis to validated mechanistic model and define the evidence base for a biomarker-selected OSCC trial of TEAD-directed combination therapy.

### 9.2. Conclusions

Cancer stem cells in OSCC are best understood not as a fixed, genetically distinct subpopulation but as a dynamic transcriptional attractor state, a stable configuration of the gene regulatory network that is self-reinforcing, resistant to displacement and continuously regenerated from the tumour populations. The persistence of this attractor state, and the failure of conventional CSC-targeting strategies to durably eliminate it, reflects the stability of the regulatory landscape that sustains it rather than the intrinsic resilience of any particular cell.

This review has proposed that three interconnected regulatory systems shape this landscape in OSCC. The Hippo–YAP pathway—genomically amplified, functionally essential, and mechanosensorily responsive—is the transcriptional stabiliser of the CSC attractor basin, sustaining nuclear YAP, activating the TAZ–TEAD4–SOX2 positive feedback circuit, and driving the mTORC1 and pEMT programmes that define the tumour-initiating cell state. Bioelectric signalling—mediated through membrane potential gradients and ion channels dynamics—acts as an upstream regulator of Hippo pathway activity, with V_mem_ depolarisation favouring YAP nuclear retention through the mechano-electro-osmotic mechanism recently established by [16] GJIC—the tissue-level integrator of individual bioelectric states—maintains the collective error-correction capacity that normally prevents cells from sustaining aberrant attractor states; its fragmentation, through connexin downregulation and post-translational channel inactivation driven by oncogenic Src/EGFR signalling, removes this protective mechanism and allows local epithelial clusters to enter the CSC basin independently of tissue-level cues.

In the context of betel quid-associated OSCC, which predominates across South and Southeast Asia—and for which no molecularly targeted prevention strategy currently exists—these three systems converge with particular biological plausibility. Betel quid exposure induces OSF, with ECM stiffening that activates Piezo1-mediated YAP nuclear translocation: arecoline generates ROS that alter ion channel function and shift V_mem_ toward depolarisation; fibrotic remodeling constrains gap junction plaque formation and fragments GJIC. Each of these is a biophysical consequence of betel quid exposure that, according to the framework developed here, contributes to deepening the CSC attractor basin before frank malignancy is clinically detectable. The OSF stage therefore represents both a mechanistic entry point into the attractor landscape and a clinical window for chemopreventive intervention, one that the experimental priorities outlined in Section 9.1 are designed to exploit.

The direct experimental evidence linking all three systems simultaneously in OSCC is absent, and this absence is the central finding of this review. It is not a weakness but a frontier. The mechanistic framework is internally coherent, grounded in primary evidence from multiple converging fields, and generates specific, falsifiable predictions that are tractable with existing experimental tools and publicly available genomic resources. Critically, because the convergence mechanism operates substantially at the post-translational level, YAP nuclear localisation, ion-channel gating, and connexin channel assembly, its predictions are most appropriately tested by assays of protein localisation and channel function rather than transcript abundance alone. A first transcript-level evaluation of the convergence predictions in public OSCC single-cell datasets is underway [84]; consistent with the post-translational nature of the proposed mechanism, it indicates that the convergence is not resolvable at the level of transcript co-regulation and reinforces the need for the protein- and functional-level assays prioritised in Section 9.1. Demonstrating that V_mem_ dysregulation alters CSC frequency in OSCC, that GJIC restoration re-engages Hippo pathway suppression, and that multi-system combination targeting achieves more durable CSC attractor displacement than single-pathway inhibition, these experiments would transform the framework from synthesis into a validated mechanistic model.

The therapeutic implication is correspondingly clear. If the CSC state in OSCC is stabilised by a convergent regulatory architecture that cannot be permanently disrupted by targeting any single component, then durable therapeutic reprogramming requires simultaneous or sequential perturbation of the landscape itself, deforming the attractor basin rather than depleting its inhabitants. The convergence node that makes this tractable is nuclear YAP: a protein whose activity is regulated by all three upstream systems, whose inhibition by TEAD-blocking small molecules is now entering clinical translation, and whose downstream transcriptional program—including mTOR activation via NRG1/AXL, pEMT programming via PDPN, and SOX2-mediated self-renewal—is now characterised at single-cell resolution in the oral epithelial progenitor cell that gives rise to OSCC. Targeting this node, informed by the bioelectric and gap junctional context that determines its upstream regulation, represents a rational and mechanistically grounded strategy for therapeutic reprogramming of OSCC that has not yet been clinically pursued. This review provides the conceptual foundation for that pursuit.

## Data Availability

No new data were created or analyzed in this study. Data sharing is not applicable to this article.

## Supplementary Materials

The following supporting information can be downloaded at: https://www.mdpi.com/article/10.3390/ijms27156649/s1.
