# Supplementary material for: A Convergence Model of Bioelectric, Gap Junctional, and Hippo–YAP Signalling in Oral Cancer Stem Cell Maintenance
Source: Int J Mol Sci. 2026 Jul 25;27(15):6649. doi: 10.3390/ijms27156649 (PMC13465928; doi:10.3390/ijms27156649)
Supplement: Supplementary file 1 [file ijms-27-06649-s001.zip › ijms-4378230-supplementary.pdf]

Supplementary Table S1. Domain classification of cited references.

Each of the 84 references cited in the review is classified as either OSCC/HNSCC-specific (studies directly investigating oral or head-and-neck squamous cell carcinoma, its potentially malignant precursors — oral submucous fibrosis and oral epithelial dysplasia — or HNSCC) or adjacent-field (sources providing mechanistic principles extrapolated from bioelectricity, connexin biology, developmental and systems biology, other cancers, or general cancer concepts). OSCC/HNSCC-specific entries are shaded.

Ref.	First author (year)	Topic / system addressed	Domain classification
1	Siegel et al. (2022)	Global cancer statistics	Adjacent — general cancer/epidemiology
2	Ghanaati et al. (2025)	OSCC recurrence after primary treatment	OSCC/HNSCC-specific — clinical
3	Hanahan & Weinberg (2011)	Hallmarks of cancer	Adjacent — general cancer concept
4	Acharya et al. (2024)	CSCs in OSCC (narrative review)	OSCC/HNSCC-specific — CSC/stemness
5	Prince et al. (2007)	CSC subpopulation in HNSCC	OSCC/HNSCC-specific — CSC/stemness
6	Gupta et al. (2011)	Stochastic state transitions (breast)	Adjacent — systems/plasticity
7	Biddle et al. (2011)	Phenotype switching in oral SCC CSCs	OSCC/HNSCC-specific — CSC/stemness
8	Vipparthi et al. (2022)	Hybrid stem-like states in oral cancer	OSCC/HNSCC-specific — CSC/stemness
9	Perez-Gonzalez et al. (2023)	Cancer cell plasticity (review)	Adjacent — systems/plasticity
10	Huang et al. (2009)	Cancer attractors (systems view)	Adjacent — systems/attractor theory
11	Nijman (2020)	Perturbation-driven entropy/heterogeneity	Adjacent — systems/attractor theory
12	Yang & Li (2025)	Atypical Hippo signalling in HNSCC	OSCC/HNSCC-specific — Hippo-YAP
13	Chai et al. (2020)	CRISPR fitness screen, Hippo in OSCC	OSCC/HNSCC-specific — Hippo-YAP
14	Li et al. (2019)	TAZ-driven SOX2 stemness in HNSCC	OSCC/HNSCC-specific — Hippo-YAP
15	Basu-Roy et al. (2015)	SOX2 antagonises Hippo (osteosarcoma/GBM)	Adjacent — Hippo mechanism (other cancer)
16	Mukherjee et al. (2026)	Vmem-Hippo mechano-electro-osmotic mechanism (MDCK/MCF10A)	Adjacent — bioelectricity (non-oral)
17	Binggeli & Weinstein (1986)	Membrane potential & growth/cancer hypothesis	Adjacent — bioelectricity
18	Levin (2021)	Bioelectric signalling (development/cancer)	Adjacent — bioelectricity
19	Yang & Brackenbury (2022)	Membrane potential in cancer	Adjacent — bioelectricity
20	Xu et al. (2023)	Fibrotic matrix to EMT in OSF (Piezo1/YAP)	OSCC/HNSCC-specific — OSF/fibrosis
21	Ko et al. (2023)	Arecoline in oral carcinogenesis	OSCC/HNSCC-specific — OSF/arecoline
22	Liu et al. (2026)	Arecoline mechanism in OSF	OSCC/HNSCC-specific — OSF/arecoline

Ref.	First author (year)	Topic / system addressed	Domain classification
23	Li et al. (2019)	YAP-induced EndMT in OSF	OSCC/HNSCC-specific — OSF/fibrosis
24	Clarke et al. (2006)	CSC perspectives	Adjacent — general CSC concept
25	Kreso & Dick (2014)	Evolution of the CSC model	Adjacent — general CSC concept
26	Yuan et al. (2019)	CancerSEA single-cell atlas (pan-cancer)	Adjacent — resource/bioinformatics
27	Mulas et al. (2021)	Cell state transitions	Adjacent — systems/plasticity
28	Jia et al. (2017)	Plasticity & dynamical systems theory	Adjacent — systems/attractor theory
29	Barkley et al. (2021)	Cancer cell states / dynamic system	Adjacent — systems/plasticity
30	Li et al. (2016)	Dynamics inside the cancer attractor (breast)	Adjacent — systems/attractor theory
31	Chiou et al. (2008)	Oct4/Nanog in oral CSCs	OSCC/HNSCC-specific — CSC/stemness
32	Yu et al. (2015)	Hippo in organ size/cancer (review)	Adjacent — Hippo mechanism (general)
33	Zhong et al. (2024)	Hippo in development/regeneration (review)	Adjacent — Hippo mechanism (general)
34	Cheng et al. (2018)	HNSCC genomic/transcriptomic characterization	OSCC/HNSCC-specific — Hippo-YAP/genomics
35	Wang et al. (2018)	Pan-cancer Hippo characterization	Adjacent — Hippo mechanism (pan-cancer)
36	Sato et al. (2025)	YAP/TAZ-TEAD targeting in HNSCC	OSCC/HNSCC-specific — Hippo-YAP
37	Overholtzer et al. (2006)	YAP 11q22 amplicon (breast)	Adjacent — Hippo mechanism (other cancer)
38	Latifian et al. (2026)	YAP in oral cancer metastasis (review)	OSCC/HNSCC-specific — Hippo-YAP
39	Omori et al. (2020)	YAP1 drives OSCC onset/progression	OSCC/HNSCC-specific — Hippo-YAP
40	Faraji et al. (2025)	YAP reprogramming of oral epithelial stem cells	OSCC/HNSCC-specific — Hippo-YAP/CSC
41	Amano et al. (2024)	Hippo protein expression in OSCC (IHC)	OSCC/HNSCC-specific — Hippo-YAP
42	Debaugnies et al. (2018)	YAP/TAZ in basal/squamous CC initiation (skin)	Adjacent — cutaneous SCC
43	Boumahdi et al. (2014)	SOX2 in squamous CC initiation (skin)	Adjacent — cutaneous SCC
44	Li et al. (2015)	TAZ to EMT/CSC in oral cancer	OSCC/HNSCC-specific — Hippo-YAP/CSC
45	Sacco et al. (2023)	SOX2 drives CSC-like phenotype, EMT/invasion, and marks OSCC close resection margins (CAL27/SCC15 + human margins)	OSCC/HNSCC-specific — CSC/stemness
46	Saw et al. (2022)	TEPD governs epithelial homeostasis	Adjacent — bioelectricity
47	Moroni et al. (2018)	Voltage gating of Piezo channels	Adjacent — bioelectricity/channel
48	Yang & Brackenbury (2013)	Membrane potential & cancer progression	Adjacent — bioelectricity
49	Srivastava et al. (2021)	Meta-analysis of bioelectric data in cancer	Adjacent — bioelectricity

Ref.	First author (year)	Topic / system addressed	Domain classification
50	Dupuy et al. (2024)	KCNA2/Kv1.2 controls YAP/Hippo (Ewing sarcoma)	Adjacent — bioelectricity/Hippo (other cancer)
51	Laird & Lampe (2018)	Connexin-targeting therapeutics (review)	Adjacent — connexin biology (general)
52	Brockmeyer et al. (2014)	Membrane Cx43 prognostic in OSCC	OSCC/HNSCC-specific — connexin/GJIC
53	Lin et al. (2001)	v-Src phosphorylation of Cx43 disrupts GJIC	Adjacent — connexin biology (general)
54	Mesnil et al. (2005)	Defective GJIC in carcinogenesis	Adjacent — connexin biology (general)
55	Camuzi et al. (2021)	HN cancer field cancerization (epigenetics)	OSCC/HNSCC-specific — clinical/field
56	Danos et al. (2015)	Cx43 prognostic value in HNSCC	OSCC/HNSCC-specific — connexin/GJIC
57	Mugundan et al. (2024)	Cx43 across OSF/OED/OSCC	OSCC/HNSCC-specific — connexin/GJIC
58	Gutierrez-Camacho et al. (2025)	Cx43 as OSCC biomarker (HPV)	OSCC/HNSCC-specific — connexin/GJIC
59	Ho et al. (2021)	Gap-junction Ca2+ in progenitor fate (Drosophila)	Adjacent — connexin/GJIC (non-oral)
60	Cervera et al. (2024)	Bioelectric network modelling	Adjacent — bioelectricity (modelling)
61	Giepmans & Moolenaar (1998)	Cx43-ZO-1 interaction	Adjacent — connexin biology (general)
62	Wilson et al. (2014)	PTPN14-Kibra-LATS1 regulates YAP	Adjacent — Hippo mechanism (general)
63	Sayedyahossein et al. (2023)	Ca2+ signalling & Hippo (review)	Adjacent — Hippo mechanism (general)
64	Azzolin et al. (2014)	YAP/TAZ in beta-catenin destruction complex	Adjacent — Hippo-Wnt mechanism (general)
65	Ghuwalewala et al. (2021)	miR-146a/AKT/beta-catenin in OSCC CSCs	OSCC/HNSCC-specific — CSC/stemness (Wnt)
66	Zhang et al. (2025)	Hippo/YAP crosstalk in cancer (review)	Adjacent — Hippo crosstalk (general)
67	Lee & Massague (2022)	TGF-beta in developmental/fibrogenic EMT	Adjacent — TGF-beta/EMT (general)
68	Sharma et al. (2025)	OSF pathogenesis (review)	OSCC/HNSCC-specific — OSF/fibrosis
69	Elkabets et al. (2015)	AXL/EGFR/PKC/mTOR resistance in HNSCC	OSCC/HNSCC-specific — PI3K/mTOR
70	Chen et al. (2020)	p53/PIK3CA in K15+ stem cells to SCC (mouse)	Adjacent — PI3K/SCC (non-oral model)
71	Gangoso et al. (2014)	c-Src/Cx43 peptide reverses glioma CSC	Adjacent — connexin/CSC (other cancer)
72	Gong et al. (2025)	Differentiation trajectories for cancer reversion	Adjacent — systems/reprogramming
73	Shin et al. (2025)	Attractor landscape in colorectal tumorigenesis	Adjacent — systems/attractor theory
74	Zeng et al. (2016)	miR-27a-3p targets YAP1 in OSCC	OSCC/HNSCC-specific — Hippo-YAP (miRNA)
75	Bian et al. (2025)	Nucleic-acid delivery systems (review)	Adjacent — drug delivery/nanotechnology

Ref.	First author (year)	Topic / system addressed	Domain classification
76	Odell et al. (2021)	Oral epithelial dysplasia grading	OSCC/HNSCC-specific — OPMD/clinical
77	Dost et al. (2014)	OED malignant transformation (grading)	OSCC/HNSCC-specific — OPMD/clinical
78	Pritzker et al. (2021)	OPMD dysplasia-grade utility	OSCC/HNSCC-specific — OPMD/clinical
79	Lin et al. (2023)	Malignant transformation of OSF	OSCC/HNSCC-specific — OSF/fibrosis
80	Shih et al. (2019)	OSF etiopathogenesis/therapy (review)	OSCC/HNSCC-specific — OSF/fibrosis
81	Zhang et al. (2022)	OSF malignant transformation (betel quid)	OSCC/HNSCC-specific — OSF/fibrosis
82	Tang et al. (2025)	OSF pathogenesis/therapeutics	OSCC/HNSCC-specific — OSF/fibrosis
83	Qin et al. (2023)	OSF mechanism/transformation/targets	OSCC/HNSCC-specific — OSF/fibrosis
84	Acharya et al. (2026)	Cross-cohort single-cell test of convergence (in prep)	OSCC/HNSCC-specific — own (in preparation)

Summary.
Of the 84 cited references, 39 (46%) are OSCC/HNSCC-specific and 45 (54%) are adjacent-field sources providing extrapolated mechanistic principles. This balance reflects the review’s cross-disciplinary aim: to integrate evidence from bioelectricity, connexin biology, and Hippo-YAP signalling — fields not previously joined in the oral-cancer context — around an OSCC-grounded convergence hypothesis.

OSCC/HNSCC-specific (39): CSC/stemness biology, 7; Hippo-YAP, 11; connexin/GJIC, 4; OSF/fibrosis and arecoline, 10; OPMD/dysplasia and clinical/field, 5; PI3K/mTOR, 1; companion study (in preparation), 1.

Adjacent-field (45): bioelectricity and ion channels, 10; connexin biology, 6; Hippo pathway mechanism and crosstalk, 9; other CSC-pathway mechanism (Wnt/TGF-β/PI3K), 2; cutaneous (non-oral) SCC, 2; systems/attractor theory and plasticity, 9; general cancer concepts, resources, other-cancer therapy and delivery, 7.
